# Oropharyngeal Lateral Wall Collapse on Drug-Induced Sleep Endoscopy as a Predictor of Hypoglossal Nerve Stimulation Response in Obstructive Sleep Apnoea—A Systematic Review

**DOI:** 10.3390/diagnostics16162529

**Published:** 2026-08-11

**Authors:** Sarah McBrinn, Oliver Skan, Aphrodite Iacovidou

**Affiliations:** 1 Imperial College NHS Foundation Trust, London W2 1NY, UK; 2 Chelsea and Westminster NHS Foundation Trust, London SW10 9NH, UK

**Keywords:** obstructive sleep apnoea, hypoglossal nerve stimulation, drug-induced sleep endoscopy, oropharyngeal lateral wall collapse, upper airway stimulation, patient selection, systematic review

## Abstract

**Background/Objectives:** Hypoglossal nerve stimulation (HNS) is an established treatment for moderate-to-severe obstructive sleep apnoea (OSA) in patients intolerant of continuous positive airway pressure. Pre-operative drug-induced sleep endoscopy (DISE) is mandatory for HNS candidacy assessment, with complete concentric velopharyngeal collapse representing the only formally accepted contraindication. The prognostic significance of oropharyngeal lateral wall (OLW) collapse remains contested, with conflicting evidence and no consensus on its role in patient selection. We sought to systematically evaluate whether OLW collapse on pre-operative DISE predicts surgical response to HNS in adults with OSA. **Methods:** A systematic review was conducted in accordance with PRISMA 2020 guidelines (PROSPERO: CRD420261347436). MEDLINE, EMBASE, and Cochrane CENTRAL were searched in March 2026 with no date restrictions. Risk of bias was assessed using the QUIPS tool. Data were synthesised narratively due to heterogeneity in DISE protocols and outcome definitions. **Results:** Eight studies comprising 1051 patients were included. Complete OLW collapse was associated with significantly lower HNS response rates in four of six studies directly testing this association (57.8–66.7% versus 74.2–84.8%; *p* = 0.024–0.042). On multivariate analysis in the largest study (*n* = 343), the association was attenuated to non-significance (OR 0.57, 95% CI 0.26–1.22), with BMI effect modification observed. One study reported a null finding and one paradoxically identified partial rather than complete OLW collapse as the failure-associated pattern (*p* = 0.04). All studies were rated as having a moderate or high risk of bias. **Conclusions:** Complete OLW collapse was associated with poorer HNS outcomes in several studies, but attenuation on multivariate analysis precludes its designation as a formal contraindication. Prospective validation and standardised DISE reporting are required before OLW collapse can inform HNS candidacy criteria.

## 1. Introduction

Obstructive sleep apnoea (OSA) is characterised by recurrent upper airway collapse during sleep and is associated with substantial cardiovascular and quality-of-life morbidity [1,2,3]. Continuous positive airway pressure (CPAP) remains the first-line therapy, but long-term adherence is frequently limited [4]. The Stimulation Therapy for Apnoea Reduction (STAR) trial demonstrated using Inspire breath synchronised stimulation led to significant improvements in objective and subjective measurements of the severity of obstructive sleep apnoea [5].

Hypoglossal nerve stimulation (HNS) is a surgical management option for the treatment of moderate-to-severe sleep apnoea [6,7]. The data supporting the efficacy of this surgical intervention includes the STAR trial and ADHERE registry real-world data, which have to date the most outcomes and data on this therapy [5]. The major advantage of this intervention is the improved adherence when compared with the use of CPAP. Currently the approved indications include, moderate-to-severe OSA, an AHI 15–65, BMI < 32 or 35 depending on the guidance, and failure of CPAP.

Drug-induced sleep endoscopy (DISE) is currently mandatory as part of the pre-operative diagnostic assessment for suitability for HNS candidacy involving pharmacological sedation and dynamic endoscopic visualisation of the upper aerodigestive tract collapse. This requires extensive standardisation to be reliable and account for variation both in conduct and interpretation [8]. The most commonly used classification system in the literature for classifying the results is the VOTE (Velum, Oropharyngeal lateral walls, Tongue and Epiglottis) system, which measures collapse at each level [9]. Complete concentric collapse (CCC) of the soft palate, identified during drug-induced sleep endoscopy (DISE), represents a key contraindication to using unilateral hypoglossal nerve stimulation (HNS) in the management of obstructive sleep apnoea (OSA) [10]. This is based on a study by Vanderveken et al. (2013) [11] which concluded that the absence of palatal CCC during DISE may predict therapeutic success with implanted UAS therapy [12]. While a widely accepted contraindication, some emerging evidence suggested that certain CCC patients may still benefit, but it remains a standard exclusion criterion [13]. The only device currently approved for use in patients with CCC is the Nyxoah Genio bilateral implant device [14].

Another common DISE finding is that of oropharyngeal lateral wall (OLW) collapse, the prevalence of which is linked to higher failure rates following surgical modification of the upper airway for treatment of OSA. This collapse often presents as a latero-lateral pattern, which stems from severe extrinsic lateral pharyngeal wall collapsibility, the presence of tonsil hypertrophy, or a combination of both [15,16]. This finding on DISE may predict failure of hypoglossal nerve stimulation, which is due to the mechanism of action of HNS to relieve the anteroposterior (AP) obstruction by stimulating the hypoglossal nerve and causing tongue protrusion. HNS primarily enlarges the airway in the anteroposterior dimension through genioglossus protrusion, which may inadequately address predominant lateral pharyngeal collapse [17,18]. Existing evidence regarding the impact of oropharyngeal lateral wall (OLW) collapse on treatment outcomes is conflicting. While some studies report poorer outcomes in the presence of OLW collapse, others demonstrate no significant association, and at least one study has described a paradoxical effect [19,20]. Consequently, there is no current consensus on its clinical significance. Although OLW collapse is not considered a formal contraindication, it is frequently considered by clinicians during patient counselling and selection. Emerging interventions, such as tonsillectomy combined with hypoglossal nerve stimulation (HNS), suggest that oropharyngeal lateral wall (OLW) collapse may be modifiable, thereby increasing the importance of clarifying its prognostic significance [19]. To date, no systematic review has specifically evaluated OLW collapse as a predictor of response to HNS.

The primary objective of this study was to systematically evaluate whether oropharyngeal lateral wall (OLW) collapse identified on pre-operative drug-induced sleep endoscopy (DISE) predicts the surgical response to hypoglossal nerve stimulation (HNS) in adults with obstructive sleep apnoea (OSA). Secondary objectives were to characterise the effect of collapse severity (complete versus partial), assess the methodological quality of the existing evidence, and identify gaps requiring prospective investigation.

## 2. Methods

### 2.1. Protocol and Registration

This systematic review was conducted and reported in accordance with the Preferred Reporting Items for Systematic Reviews and Meta-Analyses (PRISMA) 2020 guidelines. The review protocol was prospectively registered on PROSPERO (registration number: CRD420261347436) (Supplementary File S2). The scope of the review was refined by amendment in March 2026 to focus specifically on oropharyngeal lateral wall collapse as the primary DISE-derived prognostic factor of interest; this amendment was made prior to data synthesis (see Supplementary File S1).

### 2.2. Eligibility Criteria

Studies were eligible for inclusion if they enrolled adults aged 18 years or over with a confirmed diagnosis of moderate-to-severe obstructive sleep apnoea (AHI ≥ 15 events/hour); performed pre-operative drug-induced sleep endoscopy and reported oropharyngeal lateral wall collapse findings; included patients undergoing hypoglossal nerve stimulation implantation with any device; and reported a quantitative association between OLW collapse status and post-operative HNS outcome. Eligible study designs included prospective and retrospective cohort studies, case–control studies, and cross-sectional studies with outcome data. Subgroup analyses from randomised controlled trials were eligible if DISE findings and HNS outcomes were reported.

Studies were excluded if they were review articles, editorials, letters, protocols, conference abstracts without accompanying full text, or case reports with fewer than five patients. Studies were also excluded if the prognostic index test was not DISE (for example, polysomnographic airflow morphology, MRI, or cephalometry used in isolation), or if no statistical association between OLW findings and HNS outcome was reported.

### 2.3. Search Strategy

A systematic literature search was conducted in three electronic databases: MEDLINE via PubMed, EMBASE via Ovid, and the Cochrane Central Register of Controlled Trials. An original search was conducted in PubMed on 21 March 2026. A supplementary DISE-specific search was conducted in PubMed on 21 March 2026, and EMBASE via Ovid and Cochrane CENTRAL were searched on 23 March 2026. No date limits were applied. Language was restricted to English. Search terms combined three conceptual domains: hypoglossal nerve stimulation and upper airway stimulation; obstructive sleep apnoea; and drug-induced sleep endoscopy, collapse patterns, and treatment response predictors. Full search strings for each database are provided in Supplementary Table S1.

A search of ClinicalTrials.gov was conducted on 23 March 2026 to identify unpublished or ongoing studies. Two registered trials evaluating hypoglossal nerve stimulation with pre-operative DISE were identified; both were actively recruiting with no results available at the time of this review. These studies are noted as ongoing research in the discussion.

### 2.4. Study Selection

**Figure 1:**
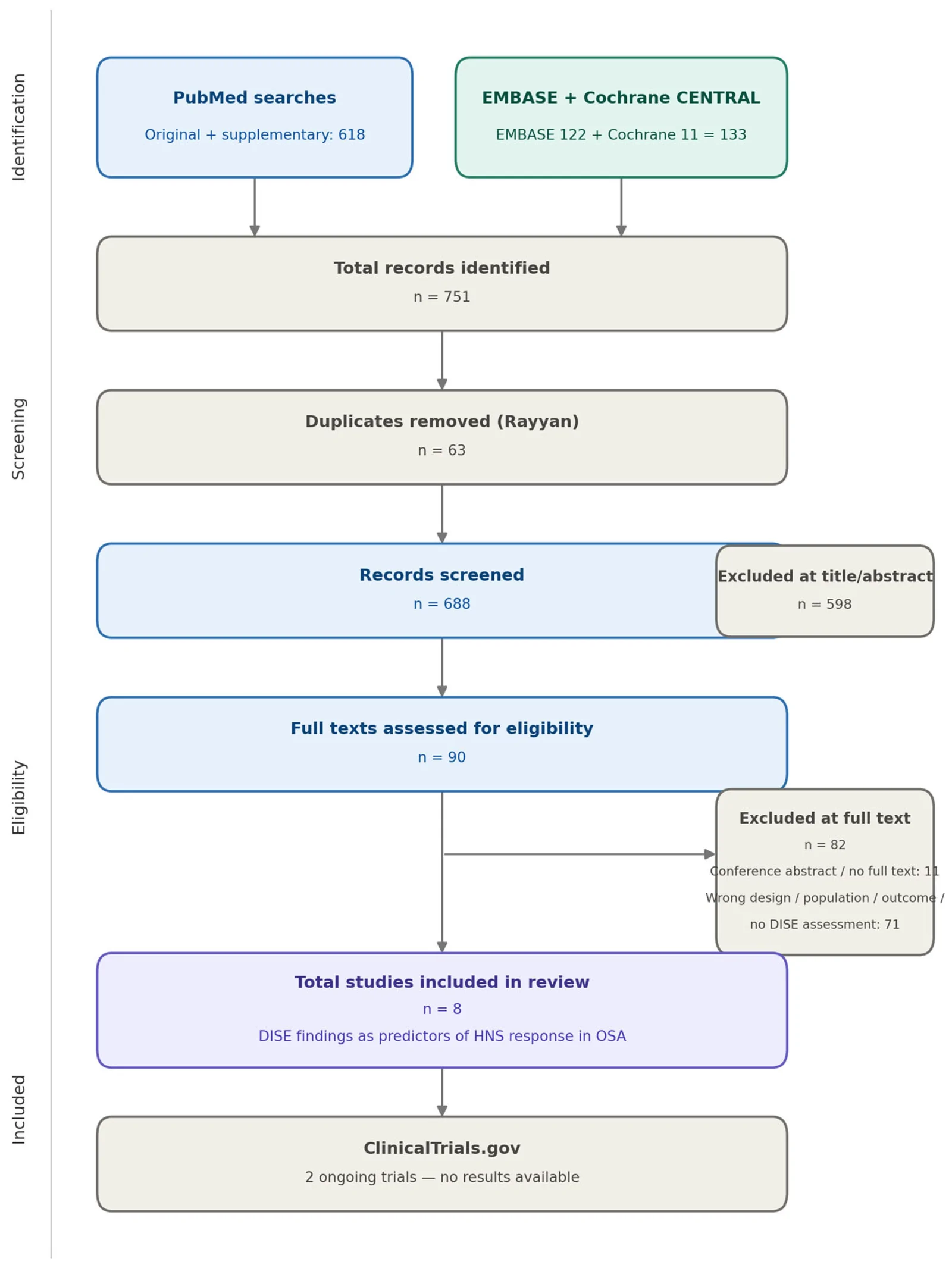
PRISMA flow diagram of the study selection process.

A flow chart of the study identification process is shown in Figure 1. Records identified from all searches were imported into Rayyan systematic review software, where duplicate records were identified and removed [21]. Title and abstract screening, followed by full-text eligibility assessment, were performed independently and in duplicate by two reviewers (S.M. and O.S.) using pre-defined inclusion and exclusion criteria. Reviewers were not blinded to authorship or journal of source. Disagreements at either stage were resolved by consensus discussion between the two reviewers, with the senior author (A.I.) available as a third reviewer to adjudicate unresolved disagreements; no third-reviewer adjudication was required. A PRISMA 2020 flow diagram was produced to summarise the study selection process (Figure 1).

### 2.5. Data Extraction

Data were extracted from included studies by the primary reviewer using a pre-specified standardised extraction form. The following variables were collected for each study: first author, year, and journal; country and number of centres; study design and data collection period; sample size including number of responders and non-responders; HNS device type and implantation criteria applied; DISE protocol including sedation agent, classification system used, and number of scorers; OLW collapse findings reported including degree and direction of collapse; statistical methods used to assess the association between OLW findings and HNS outcome; effect size and 95% confidence interval; *p*-value; surgical response definition applied; duration of follow-up; and type of post-operative sleep study [22]. Where studies reported multivariate analyses, adjusted effect estimates were extracted in preference to unadjusted estimates. Data extraction was independently verified by a second reviewer for all included studies. Discrepancies were resolved by discussion.

### 2.6. Outcome Measures

The primary outcome was HNS surgical response, defined as a reduction in AHI of ≥50% from baseline and a post-treatment AHI of <20 events/h, in accordance with the Sher criteria [21,22]. Where studies applied alternative response definitions—including AHI < 15 events/h, AHI < 10 events/h, or AHI reduction ≥ 50% without an absolute threshold—these were recorded and reported separately. Secondary outcomes included absolute AHI reduction, oxygen desaturation index reduction, and Epworth Sleepiness Scale score improvement.

### 2.7. Risk-of-Bias Assessment

The methodological quality of each included study was assessed using the Quality in Prognosis Studies (QUIPS) tool. This tool evaluates six domains: study participation, study attrition, prognostic factor measurement, outcome measurement, study confounding, and statistical analysis and reporting. Each domain was rated as low, moderate, or high risk of bias by the primary reviewer and independently verified by a second reviewer. Disagreements were resolved by discussion. An overall risk-of-bias judgement was made for each study based on the domain-level ratings.

### 2.8. Data Synthesis

Due to substantial clinical and methodological heterogeneity across included studies, formal meta-analysis was not considered appropriate. This decision was made a priori and is consistent with PRISMA 2020 guidance for systematic reviews where quantitative pooling would produce a misleading summary estimate. Sources of heterogeneity precluding meta-analysis included variation in DISE sedation protocols and sedation depth monitoring; inconsistent OLW classification systems (categorical VOTE absent/partial/complete versus continuous percentage scoring versus awake surrogate measurement); heterogeneous response definitions (standard Sher criteria AHI < 20 events/h, modified Sher criteria AHI < 15 events/h, and continuous AHI reduction without binary threshold); variation in polysomnographic outcome type (full-night in-laboratory efficacy PSG, titration PSG, home sleep apnoea testing, and mixed designs); and follow-up duration ranging from three to six months. A further source of non-combinability was the inconsistent reporting of effect measures across studies: only one study reported an odds ratio for the OLW-response association, while others reported binary success rates, continuous AHI change, or correlation coefficients, making a common effect estimate unachievable. The use of titration PSG as the primary outcome in most included studies is a recognised source of systematic bias, as titration PSG overestimates treatment response compared to full-night efficacy PSG. Pooling response rates derived from these methodologically distinct outcome measures would introduce systematic error of uncertain direction and magnitude into any summary estimate. Data were therefore synthesised narratively. Studies were grouped according to the direction and statistical significance of their reported association between OLW collapse and HNS response, and a direction of effect table was constructed to present findings in a standardised format. The overall certainty of evidence was assessed using the GRADE framework as described in Section 2.8.

## 3. Results

### 3.1. Search Results and Study Selection

Database searches conducted in March 2026 identified 751 records across MEDLINE (*n* = 618, including original and supplementary searches), EMBASE via Ovid (*n* = 122), and Cochrane CENTRAL (*n* = 11). A further two records were identified via ClinicalTrials.gov; both represented ongoing recruiting trials with no results available and were therefore excluded from the primary synthesis. Following removal of 63 duplicates using Rayyan, 688 unique records were screened at the title and abstract level, of which 598 were excluded. Ninety full texts were assessed for eligibility; 82 were excluded at this stage, comprising 11 conference abstracts for which no full text was retrievable, and 71 records excluded for wrong study design, population, outcome, or absence of DISE data. Eight studies met all eligibility criteria and were included in the primary synthesis. The selection process is summarised in the PRISMA 2020 flow diagram (Figure 1).

### 3.2. Characteristics of Included Studies

The eight included studies were published between 2013 and 2025 and reported data from a combined total of 1051 patients (range 21–369 per study; noting partial overlap between Huyett 2021 and Huyett 2024 registry populations) [19,23]. Study characteristics are summarised in Table 1. Total reflects the analytic cohort across studies. Where studies reported multiple denominators (e.g., DISE cohort versus implanted versus follow-up cohort), the cohort contributing data to the OLW–response analysis was used. Vena 2025: *n* = 369 in the DISE cohort (Aim 1) [17]. Partial methodological and cohort overlap may exist between Huyett et al. 2021 and Huyett et al. 2024 [19,23], both of which originated from Massachusetts Eye and Ear and shared overlapping recruitment periods. However, the studies differed in design, inclusion criteria, and primary analytic aims, with Huyett 2024 specifically evaluating concomitant tonsillectomy in OLW-positive patients [19].

Five studies were retrospective cohort designs and three were prospective: Mulholland and Dedhia (2020) was a prospective case series, Huyett et al. (2024) was a prospective observational case–control study, and Vena et al. (2025) was a prospective observational cohort study [17,19,24]. Eight studies were conducted at single centres; Huyett et al. (2021) [23] was the sole multicentre study, drawing on data from 10 academic centres across the United States and Germany. Sample sizes ranged from 21 (Vanderveken 2013) to 369 (Vena 2025) [11,17]. All studies used the Inspire upper airway stimulation system, and no studies reported outcomes for alternative HNS devices. Studies were conducted in the United States (*n* = 6), Germany (*n* = 1, Pordzik 2023), and Belgium/Netherlands/Germany multicentre (*n* = 1, Vanderveken 2013) [11,25].

Follow-up duration ranged from approximately three months (Pordzik 2023, Nord 2024) to six months (Vanderveken 2013, Mulholland 2020) [11,16,24,25]. Post-operative outcome assessment varied considerably: five studies used titration polysomnography (tPSG) as the primary outcome measure (Vanderveken 2013, Huyett 2021, Huyett 2024—the last using entire-night tPSG AHI explicitly justified as more conservative than optimal-setting AHI); three used a mixture of tPSG and home sleep apnoea testing (HST; Chao 2020, Nord 2024, Vena 2025); and two used full-night in-laboratory PSG (Mulholland 2020 efficacy PSG; Pordzik 2023 AASM full-night PSG) [11,13,16,17,19,23,24,25].

### 3.3. DISE Protocols and OLW Classification

Sedation protocols varied across included studies. Propofol was the predominant sedative agent, administered using several different protocols including target-controlled infusion, probability ramp infusion, microbolus, and weight-based infusion. The VOTE classification was employed in seven of eight studies; Vanderveken 2013 used a pre-VOTE bespoke system scoring level, direction, and degree of collapse. OLW collapse was graded categorically as absent, partial, or complete in seven studies and as a continuous percentage in one (Pordzik 2023, mean OPLW 16.8 ± 17.4%) [11,25].

The number of DISE scorers varied substantially. Huyett 2021 used four blinded reviewers with a priori consensus criteria and reported formal inter-rater reliability (κ = 0.48 for OLW degree, moderate agreement) [23]. The remaining studies used a single unblinded scorer—in most cases, the operating surgeon—with no inter-rater reliability reported; this was explicitly acknowledged as a limitation in Vena 2025. DISE videos were recorded and available for review in Huyett 2021, and Mulholland 2020 explicitly stated that DISE was not recorded [23,24].

### 3.4. Prevalence of OLW Collapse Across Studies

Where reported, the prevalence of any oropharyngeal lateral wall collapse ranged from 14.3% (Vanderveken 2013, classified using a pre-VOTE system in which 35.1% had any form of lateral collapse) to 100% (Huyett 2024, OLW collapse being an inclusion criterion by design) [11,19]. In Nord 2024, the largest dedicated OLW study (*n* = 111), 40.5% had complete, 27.0% partial, and 32.4% no OLW collapse [11,19]. In Huyett 2021 (*n* = 343), OLW degree distribution was absent 64.1%, partial 26.2%, and complete 9.6% [23]. In Chao 2020 (*n* = 68), oropharyngeal collapse distribution across the whole cohort was none 76.5%, partial 19.1%, and complete 4.4% [13]. In Vena 2025 (*n* = 330 analytic cohort), complete OLW collapse was identified in 27 patients (8.2%), the lowest prevalence among the dedicated OLW studies and reflecting the strict eligibility criteria for HNS candidacy at the recruiting centre [17].

### 3.5. Association Between OLW Collapse and HNS Response

Quantitative meta-analysis was not performed for the reasons outlined in Section 2.8. Findings were therefore synthesised narratively, organised by direction of effect, and supplemented by a structured direction-of-effect summary (Table 2). Formal assessment of reporting bias by a funnel plot was not feasible in the absence of a pooled effect estimate; potential reporting bias was instead considered narratively in the context of industry funding ties (Section 3.6) and is discussed in Section 4. Pre-specified subgroup analyses by BMI and tonsil size were not feasible due to inconsistent reporting of these covariates across studies; sensitivity analyses were correspondingly not performed.

#### 3.5.1. Studies Reporting a Significant Negative Association

Four studies reported a significant association between OLW collapse and reduced HNS response, presented below in chronological order of publication. These studies span the methodological spectrum of the included evidence base, from a small prospective case series (Mulholland and Dedhia 2020, *n* = 46) to the largest prospective cohort in the field (Vena 2025, *n* = 369) and together provide the most direct evidence on the possible prognostic significance of complete OLW collapse [17,24].

Mulholland and Dedhia (2020) reported the first dedicated analysis of baseline OLW collapse as a predictor of HNS response using continuous AHI reduction as the outcome [24]. Patients with complete baseline lateral wall collapse (*n* = 14) demonstrated significantly less AHI reduction than those with partial collapse (*n* = 32): mean AHI reduction 9.0 (95% CI −2.3 to 20.3) versus 22.4 (95% CI 14.9 to 29.9) events/h respectively (*p* = 0.05). This study was the only included study to use full-night in-laboratory efficacy PSG as the outcome measure, representing a methodological strength [24].

Huyett et al. (2021) provided one of the most rigorously conducted analyses prior to Vena 2025, with 343 patients across 10 centres and four blinded DISE reviewers [17,24]. Complete OLW collapse was associated with a lower treatment response rate compared to partial or absent OLW collapse: 57.8% versus 74.2% (*p* = 0.042) using the primary definition of AHI reduction ≥ 50% to <15 events/h on tPSG. Notably, there was no significant difference in mean AHI change between OLW groups (*p* = 0.94), suggesting OLW collapse affected the likelihood of meeting a binary threshold rather than the magnitude of continuous AHI improvement. On multivariable analysis adjusting for age, sex, BMI, and baseline AHI, this association was attenuated to non-significance (adjusted OR 0.57, 95% CI 0.26–1.22, *p* > 0.05). A significant BMI-by-OLW interaction was noted: the negative association between complete OLW collapse and HNS response remained significant in patients with BMIs above the cohort median but was not significant below it.

Nord et al. (2024) conducted the only study with lateral wall collapse as the primary and sole outcome of interest, in a single-centre cohort of 111 patients [16]. Patients with complete OLW collapse (*n* = 45) had a significantly lower surgical success rate than those with partial or no collapse (*n* = 66): 66.7% versus 84.8% (*p* = 0.024) using modified Sher criteria (AHI < 15 events/h). Baseline demographics including age, BMI, sex, and pre-operative AHI were not significantly different between groups, supporting OLW collapse degree as an independent explanatory variable. However, no multivariate analysis was performed. Importantly, the association appeared binary rather than graded: partial OLW collapse was not associated with worse outcomes compared to no collapse (90.0% versus 80.6% success), a finding that directly conflicts with Chao and Thaler (2020) [13].

Vena et al. (2025) provided the largest and methodologically strongest assessment to date of OLW collapse as a predictor of HNS efficacy [17]. In a prospective single-centre cohort of 369 HNS candidates undergoing DISE, of whom 330 entered the descriptive analysis after exclusion of 39 patients with concomitant or prior upper airway surgery, complete OLW collapse was identified in 27 patients (8.2%). Notably, partial OLW collapse was modelled as a separate covariate and was not significantly associated with reduced efficacy, mirroring the binary pattern reported by Nord et al. (2024) and contrasting with the partial-collapse association reported by Chao and Thaler (2020) [13,16]. This study addresses several methodological limitations of prior work: prospective design, the largest dedicated sample size in the field, pre-specified analyses, and multivariable adjustment for established confounders including PSG type. Limitations include single-centre recruitment at a tertiary academic centre with extensive industry funding ties, a single-surgeon DISE scoring protocol without inter-rater reliability assessment, and short-term follow-up.

#### 3.5.2. Studies Reporting Null, Conflicting or Indirect Findings

Three studies did not provide direct evidence of a negative association between complete OLW collapse and HNS response. These comprised two studies in which OLW was not formally tested as an independent predictor (Vanderveken 2013, Mahmoud and Thaler 2019), one study reporting a paradoxical association (Chao and Thaler 2020), one study reporting null findings on continuous AHI outcomes despite a weak association with snoring (Pordzik 2023), and one study providing indirect support via an awake clinical surrogate (Weiner 2025) [11,13,25,26].

Vanderveken et al. (2013), the foundational study establishing CCC as a contraindication, noted oropharyngeal lateral collapse in 14.3% of their cohort (*n* = 3/21) but performed no formal statistical analysis of OLW as an independent predictor. This study was underpowered for this analysis, and OLW was not a primary outcome [11].

Chao and Thaler (2020) reported a paradoxical and clinically important finding: partial—not complete—OLW collapse was significantly associated with treatment failure [13]. All three patients with complete OLW collapse responded successfully to HNS, though this subgroup was critically underpowered (*n* = 3) to draw meaningful conclusions. The authors postulated that lateral oropharyngeal collapse may be physiologically analogous to complete concentric palatal collapse, with palatopharyngeus hyperactivity as a potential common mechanism.

Pordzik et al. (2023) [25] assessed OLW collapse as a continuous percentage variable using Spearman’s correlation in 25 patients and found no significant correlation between OPLW degree and post-operative AHI. The only significant OLW finding was a weak positive correlation between OPLW degree and post-operative snoring index, suggesting residual lateral wall vibration may persist as a symptom even when AHI responds to HNS. The primary positive predictor in this study was tongue base obstruction, which strongly correlated with AHI reduction. The mean OPLW obstruction degree of 16.8% in this cohort was notably low, limiting statistical power to detect an OLW-specific effect.

#### 3.5.3. OLW Collapse as a Therapeutic Target

One study investigated OLW collapse not as a fixed prognostic factor but as a potentially modifiable target. Rather than testing whether OLW collapse predicts HNS response, this study examined whether concomitant surgical treatment of the lateral wall could mitigate its adverse effect, addressing a distinct but complementary research question to the prognostic studies reviewed above.

Huyett et al. (2024) investigated a novel surgical strategy to mitigate the adverse prognostic effect of OLW collapse [19]. In a prospective case–control study of 97 patients, all of whom had partial or complete OLW collapse and small tonsils (Brodsky grade 1+ or 2+), concomitant palatine tonsillectomy and HNS (HNS + T, *n* = 19) was compared against HNS alone (*n* = 78). On multivariable linear regression adjusting for OLW severity, tonsil size, age, sex, BMI, and baseline AHI, HNS + T was associated with an additional 22.9% (95% CI 7.5–35.2%) reduction in entire-night AHI compared to HNS alone (*p* = 0.006), and 8.6-fold greater odds of a successful treatment response (95% CI 1.7–43.4, *p* = 0.010). These results were consistent across alternative response definitions and optimal-setting AHI analyses. The procedure added a mean of 17 min to operating time, and post-tonsillectomy haemorrhage occurred in 2 of 30 patients (6.7%). No infections were reported. These early results suggest tonsillectomy may partially offset the negative prognostic impact of OLW collapse, with HNS + T response rates (79%) approximating those of non-OLW collapse patients in the same registry (86%).

### 3.6. Risk of Bias

Risk of bias was assessed using the QUIPS tool across six domains (see Table 3). The overall risk of bias was rated as moderate in four (Mulholland and Dedhia 2020, Huyett 2021, Huyett 2024, Vena 2025) and high in six (Vanderveken 2013, Chao and Thaler 2020, Pordzik 2023, Nord 2024) [11,13,16,17,23,24,25,27].

The two domains driving most high-risk ratings were study confounding and prognostic factor measurement. Five of eight studies provided no multivariable adjustment for established confounders of HNS response—including BMI, baseline AHI, age, and tonsil size—and reported only unadjusted comparisons between OLW collapse strata. Only Huyett 2021, Huyett 2024, and Vena 2025 reported multivariable analyses, and only Vena 2025 pre-specified the OLW–response model with adjustment for the full set of established confounders [17,19,23]. Huyett 2021 was the sole study to use multiple blinded DISE reviewers with a published inter-rater agreement statistic (κ = 0.48 for OLW degree) [23].

Outcome measurement was rated moderate or high in all eight studies, reflecting widespread reliance on titration polysomnography rather than full-night efficacy studies, and acknowledged within several included papers as a methodological limitation. Mulholland and Dedhia 2020 and Pordzik 2023 were the only studies to use full-night in-laboratory PSG as the primary outcome measure [24,25]. Study participation and attrition domains were generally rated moderate or low across studies, with sample size and single-centre recruitment the most frequent participation concerns.

Vena 2025 represented the methodologically strongest evidence in the body of literature reviewed, by virtue of prospective design, the largest dedicated sample size in the field, pre-specified multivariable adjustment for established confounders, and reporting in accordance with prognosis-study standards; its principal residual limitations were single-centre recruitment, a single-surgeon DISE scoring protocol without inter-rater reliability data, and short-term follow-up [17]. Huyett 2021 retained methodological strengths complementary to Vena 2025, particularly its multicentre design and formal inter-rater reliability assessment of OLW grading [17,23].

Industry funding or author consultancy with Inspire Medical Systems was disclosed in the majority of studies. While not a formal QUIPS domain, this represents a relevant consideration for interpretation of effect sizes and is noted in Table 3. Per-study domain ratings and supporting rationale are provided in Table 3.

### 3.7. Certainty of Evidence

Certainty of evidence was assessed using the GRADE framework adapted for prognostic factor research, with certainty judged across relevant domains rather than assuming an automatic high certainty starting point. The evidence was downgraded for risk of bias (−1), as four of the eight studies were rated high risk of bias and the remainder, including the largest prospective cohort (Vena 2025), were rated moderate risk [17]. Certainty was also downgraded for inconsistency (−1), reflecting conflicting findings across studies, including paradoxical and null associations, as well as variation between partial and complete OLW collapse patterns. Indirectness (−1) was applied because Sher response is a surrogate rather than a patient-centred outcome, and most studies relied on titration polysomnography rather than full-night efficacy assessment.

Imprecision was not downgraded, as the combined analytic cohort included 1051 patients across ten studies, including the largest dedicated prospective cohort in the field (Vena 2025, *n* = 369), which demonstrated a statistically significant adjusted effect estimate [17]. Publication bias could not be formally assessed in the absence of meta-analytic pooling; while industry funding ties in the majority of studies represent a relevant consideration, the consistency of direction of effect across studies of varying funding sources mitigates against a serious publication bias concern at this stage.

## 4. Discussion

### 4.1. Summary of Principal Findings

This systematic review synthesised evidence to evaluate whether oropharyngeal lateral wall collapse on pre-operative drug-induced sleep endoscopy predicts hypoglossal nerve stimulation response in adults with obstructive sleep apnoea. Among the studies directly evaluating OLW collapse as a predictor, complete OLW collapse was associated with reduced HNS response, with surgical success rates of 57.8–66.7% in patients with complete OLW collapse compared to 74.2–84.8% in patients without complete OLW collapse. The largest and methodologically strongest study, Vena et al. 2025 (*n* = 369), reported an 18.0% smaller adjusted reduction in apnoea–hypopnoea index after multivariable regression (95% CI −31.9 to −6.2, *p* = 0.002) [17]. The relationship between collapse severity and treatment outcome is inconsistent across studies, with some suggesting a graded effect and others a binary threshold, with partial OLW collapse generally not associated with reduced response. Emerging evidence from Huyett et al. 2024 indicates that concomitant tonsillectomy may partially mitigate the adverse prognostic effect of OLW collapse, reframing it as a potentially modifiable surgical target [19]. Overall certainty of evidence was rated LOW.

### 4.2. Interpretation in Context of Existing Evidence

The findings of this review build on the patient selection paradigm established by the STAR trial and the Vanderveken 2013 cohort, in which complete concentric velopharyngeal collapse was identified as the principal contraindication to HNS [9]. Complete OLW collapse has traditionally been considered a relative rather than absolute contraindication, and the present synthesis suggests it may also adversely affect treatment response. Across studies directly assessing this relationship, complete OLW collapse was associated with approximately 15–20% lower response rates, with Vena 2025 demonstrating a persistent adjusted effect after accounting for established confounders [17]. However, heterogeneity and predominantly retrospective study designs limit definitive conclusions regarding its role in formal patient selection.

A methodological caveat applies to the concordance of findings across studies: Huyett 2021 and Huyett 2024 both report a negative direction of effect for OLW collapse, but they draw on partially overlapping registry populations from the same institution [19,23]. Because these are not fully independent samples, their agreement should not be read as replication by two separate patient cohorts; the effective independent evidence base for the negative association is smaller than the raw study count of four (Mulholland, Huyett 2021, Nord, Vena) suggests, and this partial non-independence has been factored into the inconsistency downgrade in the GRADE assessment (Section 3.7) rather than treated as reinforcing certainty [16,17,23,24].

### 4.3. Methodological Considerations and Sources of Heterogeneity

Substantial methodological heterogeneity limits comparability across studies. Only Huyett 2021 reported formal inter-rater reliability for OLW grading (κ = 0.48, indicating moderate agreement), highlighting concerns about DISE reproducibility and standardisation [9,23]. The VOTE classification used in nine of ten studies reduces a continuous anatomical phenomenon to a three-level ordinal grade, and no study reported a formal definition for the cut-off between collapse categories. Sedation protocols also varied considerably, with bi-spectral index monitoring reported in only three studies. Outcome assessment further contributed to heterogeneity, as approximately half of studies used titration polysomnography rather than full-night efficacy testing, which may overestimate residual AHI [28]. Reported OLW prevalence varied widely (8.2–40.5%), likely reflecting differences in patient selection and centre-level candidacy thresholds rather than true epidemiological variation.

The BMI-by-OLW interaction observed in Huyett 2021 is arguably the most important effect-modifying finding in this evidence base [23]. Mechanistically, higher adiposity is associated with greater parapharyngeal fat deposition and lateral pharyngeal wall thickness, which may compound the structural narrowing already produced by OLW collapse—potentially explaining why HNS, which acts primarily on anteroposterior tongue-base obstruction, fails more consistently in this subgroup. This raises the possibility that BMI does not merely confound the OLW–response relationship but actively modifies it, meaning future studies should report OLW findings stratified by BMI rather than adjusting for it as a simple covariate.

### 4.4. Clinical Implications

The available evidence supports consideration of complete OLW collapse on DISE as a relative—not absolute—contraindication to HNS. Although surgical success rates are reduced in patients with complete OLW collapse, a proportion of these patients still meet response criteria, and alternatives may be limited in patients intolerant of continuous positive airway pressure and unsuitable for maxillomandibular advancement. Outright exclusion from HNS candidacy on the basis of complete OLW collapse alone is therefore not justified by the current evidence.

These findings may inform pre-operative counselling. Patients identified with complete OLW collapse on DISE may be counselled that observed response rates can be approximately 15–20 percentage points lower than those without complete collapse, and the implications of this for informed consent should be documented.

Where complete OLW collapse is identified in patients with small palatine tonsils (Brodsky grade 1+ or 2+), the evidence from Huyett suggests that concomitant tonsillectomy may warrant consideration, with reported success rates approaching those of OLW-negative patients in the same registry [19,28]. This intervention adds modest operative time and carries a small post-tonsillectomy haemorrhage risk but represents the only currently described surgical strategy with prospective evidence of OLW-effect mitigation. For patients with complete OLW collapse who are not tonsillectomy candidates or in whom adjunctive intervention is declined, multidisciplinary discussion involving sleep medicine, otolaryngology, and oral and maxillofacial surgery is warranted to evaluate alternative or combination therapeutic strategies.

### 4.5. Future Research Priorities

The LOW certainty of evidence reflects specific, addressable methodological gaps that define the priorities for future research. Standardisation of DISE reporting protocols, including pre-specified criteria for partial versus complete OLW grading and routine reporting of inter-rater reliability, is the most fundamental need. The 2018 European DISE position paper provides a starting framework but has not been operationalised in the HNS literature [9]. If reproducible, non-invasive identification of OLW collapse from polysomnographic flow morphology would enable pre-DISE risk stratification and could supplement DISE-based grading [17]. Multicentre prospective registries with pre-specified OLW-stratified analyses, BMI interaction terms, and full-night efficacy outcomes would address several of the heterogeneity sources identified in the present synthesis.

The Huyett 2024 finding warrants confirmation in a randomised design [19]. A randomised trial of HNS plus concomitant tonsillectomy versus HNS alone in OLW-positive patients with small tonsils would establish whether the observed mitigation effect is causal and would directly inform patient selection criteria. Long-term follow-up beyond the three-to-twelve-month windows that dominate the current literature, with patient-centred outcomes including symptom resolution, device adherence, and cardiovascular endpoints, is similarly required.

Finally, none of the included studies tested non-Inspire HNS devices. As bilateral and alternative-design HNS systems gain regulatory approval and clinical adoption, replication of the OLW–response association across device platforms will be necessary to establish whether the present findings represent a general phenomenon or are specific to unilateral genioglossus stimulation.

### 4.6. Strengths and Limitations of This Review

This review has several methodological strengths. The protocol was prospectively registered with PROSPERO (CRD420261347436), and the search strategy spanned three databases supplemented by clinical-trial registry searching. Title, abstract, and full-text screening were conducted independently by two reviewers with consensus adjudication. Inter-rater agreement for screening decisions was substantial, Cohen’s κ = 0.61. Risk of bias was assessed at the domain level using the QUIPS tool, and certainty of evidence was rated using a GRADE framework adapted for prognostic factor reviews.

Several limitations should be acknowledged. The search was restricted to English-language publications. This is a significant limitation given that DISE protocols and classification systems have been extensively developed and reported by European groups; non-English-language studies, particularly from continental Europe, may have been systematically excluded, potentially biasing the evidence base toward North American practice patterns and case mix. Grey literature beyond ClinicalTrials.gov was not systematically searched; relevant non-English or non-indexed studies may have been missed. Substantial clinical and methodological heterogeneity precluded quantitative meta-analysis, and narrative synthesis carries an inherent element of subjective judgement in weighting findings across studies of varying quality.

Partial registry overlap exists between Huyett 2021 and Huyett 2024, both drawing on Massachusetts Eye and Ear datasets; this may reduce the apparent independence of evidence from these two studies [19,23]. Industry funding or author consultancy with the device manufacturer was disclosed in the majority of included studies and could not be quantitatively addressed in the absence of meta-analytic pooling. Finally, most included studies were conducted at single centres in the United States; applicability of these findings to other healthcare contexts and patient populations remains uncertain.

Current evidence supports consideration of complete oropharyngeal lateral wall collapse as a relative negative prognostic factor warranting discussion during HNS candidacy counselling but does not justify its incorporation as a formal exclusion criterion at present. Standardisation of DISE reporting protocols, adoption of full-night efficacy polysomnography as the primary outcome measure, and prospective multicentre validation studies remain the principal research priorities.

Industry funding or author consultancy with Inspire Medical Systems was disclosed in the majority of included studies, which warrants explicit consideration given the commercial context: OLW collapse findings on DISE directly inform HNS candidacy decisions for the manufacturer’s device. This creates a theoretical incentive structure in which studies could be more likely to characterise OLW collapse as a modifiable or non-absolute barrier to candidacy (as in Huyett 2024) than as a hard exclusion [19]. We note, however, that the direction of effect (negative association between complete OLW collapse and response) was consistent across studies regardless of funding disclosure, which offers some reassurance against differential reporting bias. This could not be formally tested in the absence of meta-analytic pooling, and independent, non-industry-funded validation of the OLW–response association remains an important priority.

## 5. Conclusions

Complete oropharyngeal lateral wall collapse on drug-induced sleep endoscopy may contribute to a reduced response to hypoglossal nerve stimulation, though the association was not independently sustained after multivariable adjustment in the largest and most methodologically robust study to date. The relationship between collapse severity and treatment outcome is inconsistent across studies, with some suggesting a graded effect and others a binary threshold at complete collapse, reflecting the limitations of current VOTE classification systems. Emerging evidence suggests that the effect of complete lateral wall collapse may be partially mitigated by concomitant palatine tonsillectomy in eligible patients with small tonsils, though this requires prospective confirmation. Overall certainty of evidence is low, reflecting predominantly retrospective single-centre study designs, substantial methodological heterogeneity, and reliance on titration polysomnography as a surrogate response measure in most included studies.

## Figures and Tables

**Table 1:** Characteristics of included studies. (**A**) Study design and cohort characteristics. (**B**) DISE protocol and outcome assessment.

**(A)**												
**Study**	**Country**	**Design**		**Centres**	**Period**		***N* (** **R** **:** **NR** **)**		**Age (yrs)**		**BMI (kg/m^2^)**	
Vanderveken 2013 [11]	Belgium/Netherlands/Germany	Retrospective cohort		1	Pre-2012		21 (13:8)		55 ± 11		28 ± 2	
Mulholland and Dedhia 2020 [24]	USA	Prospective case series		1	October 2015–January 2019		46 (NR)		64.4 ± 15.2		28.5 ± 3.7	
Chao and Thaler 2020 [13]	USA	Retrospective chart review		1	2015–2018		68 (54:14)		Success 62.5 ± 10.5; Failure 55.2 ± 10.0		Success 29.8 ± 4.2; Failure 29.4 ± 3.0	
Huyett 2021 [23]	USA	Retrospective multicentre cohort		10	Pre-2020 (NR)		343 (249:94)		60.4 ± 11.0		29.2 ± 3.6	
Pordzik 2023 [25]	Germany	Retrospective single-centre cohort		1	February 2020–December 2022		25 (NR)		54.5 ± 9.6		30.0 ± 4.0	
Nord 2024 [16]	USA	Retrospective single-centre cohort		1	January 2014–June 2022		111 (86:25)		63.6 ± 11.4		29.3 ± 3.2	
Huyett 2024 [19]	USA	Prospective case–control		1	November 2020–October 2023		97 (19 + T; 78 control)		HNS + T 55 (48–61); control 59 (53–65)		HNS + T 31.0 (27.9–31.8); control 29.8 (26.7–31.3)	
Vena 2025 [17]	USA	Prospective observational cohort		1	October 2019–July 2023		369 DISE; 330 analysed (191:139)		Median 61 (IQR 53–68)		Median 28.8 (IQR 26.5–30.8)	
**(B)**												
**Study**	**OLW Classification**		**OLW Prevalence**			**Response Definition**		**Overall Response**		**Follow** **-** **Up**		**PSG Modality**
Vanderveken 2013 [11]	Pre-VOTE bespoke		Lateral collapse 14.3%			Sher: AHI < 20 + ≥50% reduction		62% (13/21)		6 months		Full night in laboratory
Mulholland and Dedhia 2020 [24]	VOTE ± bespoke 0/1/2 MA grading		Complete OLW 14/46 (30.4%); partial 32			Continuous AHI change		AHI 39.4 → 21.1 (*p* < 0.01)		~6 months		Full night efficacy
Chao and Thaler 2020 [13]	VOTE		Complete 4.4%; partial 19.1%; none 76.5%			Sher: AHI < 20 + ≥50% reduction		79.4% (54/68)		Variable		Mixed tPSG/HST/full night
Huyett 2021 [23]	VOTE; 4 blinded reviewers; κ = 0.48		Complete 9.6%; partial 26.2%; absent 64.1%			Modified Sher: AHI < 15 + ≥50% reduction		72.6% (tPSG); 57.3% (HSAT)		4.1 ± 4.1 mo (tPSG); 16.5 ± 10.8 mo (HSAT)		Titration; HSAT subgroup
Pordzik 2023 [25]	VOTE continuous %		Mean OPLW 16.8 ± 17.4%			Continuous correlations (Spearman)		AHI 35.6 → 22.6 (~37% reduction)		~3 months		Full night in-laboratory
Nord 2024 [16]	VOTE 0/1/2; video-recorded		Complete 40.5%; partial 27.0%; none 32.4%			Modified Sher: AHI < 15 + ≥50% reduction		77.5% (86/111)		~3 months		Mixed all night HST/tPSG
Huyett 2024 [19]	VOTE		All OLW-positive (inclusion criterion)			Modified Sher: AHI < 15 + ≥50% reduction (entire night)		HNS + T 79%; control 48.9%		Mean 160 days (70–251)		Titration (entire night AHI)
Vena 2025 [17]	VOTE		Complete 27/330 (8.2%)			Modified Sher: AHI < 15 + ≥50% reduction; continuous % AHI reduction		57.9%		≥3 months post-activation		Mixed: tPSG 78%; HSAT 22%

R, responder; NR, non-responder. R:NR breakdown reflects the analytic cohort reported by the source study; derived figures are noted in the text. See Table 1B for DISE protocol and outcome details, and the manuscript text for full denominator notes. **Abbreviations:** BMI, body mass index; CI, confidence interval; HNS + T, hypoglossal nerve stimulation plus concomitant tonsillectomy; IQR, interquartile range; NR, not reported; SD, standard deviation; R, responder; NR, non-responder; AHI, apnoea–hypopnoea index; CCC, complete concentric collapse; DISE, drug-induced sleep endoscopy; HNS, hypoglossal nerve stimulation; HSAT, home sleep apnoea test; HST, home sleep test; MA, multilevel airway; OLW, oropharyngeal lateral wall; OPLW, oropharyngeal lateral wall (continuous percentage); PSG, polysomnography; SPW, supine pharyngeal width; tPSG, titration polysomnography; VOTE, Velum/Oropharynx/Tongue base/Epiglottis classification. R:NR breakdown reflects analytic cohort reported by the source study; derived figures are noted in the text. Huyett 2024 [19]: case–control comparing HNS plus concomitant tonsillectomy (*n* = 19 with follow-up of 30 procedures performed) versus HNS alone in OLW-positive patients (*n* = 78 control). Vena 2025 [17]: DISE cohort *n* = 369; 39 patients with concomitant or prior upper airway surgery excluded from descriptive denominators leaving *n* = 330; Aim 2 cohorts (DISE Flow *n* = 138; HSAT validation *n* = 46) reported separately in source.

**Table 2:** Summary of direction of effect of OLW collapse on HNS response across included studies.

Study	*N*	OLW Classification	Comparison	Direction of Effect	Statistically Significant	PSG Type	Overall RoB
Vanderveken 2013 [11]	21	Pre-VOTE: latero-lateral	OLW 14.3%—no formal comparison	Not tested	N/A	Full night in lab	HIGH
Mulholland and Dedhia 2020 [24]	46	VOTE categorical	Complete vs. partial OLW → less AHI reduction	↓ Negative	Yes (*p* = 0.05)	Full night efficacy	MODERATE
Chao and Thaler 2020 [13]	68	VOTE categorical	Partial OLW → failure; complete OLW (*n* = 3) all succeeded	↓ Paradoxical	Yes (*p* = 0.04)	Mixed tPSG/HST	HIGH
Huyett 2021 [23]	343	VOTE categorical	Complete vs. partial/none → lower response; NS on multivariate	↓ Negative (univariate only)	Yes univariate (*p* = 0.042); NS multivariate (*p* > 0.05)	Titration	MODERATE
Pordzik 2023 [25]	25	VOTE continuous %	OLW degree—no correlation with AHI outcome	→ Null	No (*p* = 0.11–0.85)	Full night in lab	HIGH
Nord 2024 [16]	111	VOTE categorical	Complete vs. partial/none → lower success rate	↓ Negative	Yes (*p* = 0.024)	Mixed tPSG/HST	HIGH
Huyett 2024 [19]	97	VOTE categorical	All patients OLW collapse—tonsillectomy mitigates effect	↓ Negative (presupposed)	N/A—intervention study	Titration	MODERATE
Vena 2025 [17]	369 (330 analysed)	VOTE categorical	Complete vs. no OLW → smaller AHI reduction (adjusted)	↓ Negative	Yes (*p* = 0.002, multivariate)	Mixed tPSG (78%)/HSAT (22%)	MODERATE

**Table 3:** Risk-of-bias assessment using the QUIPS tool across six domains, with overall rating and supporting rationale.

Study	Participation	Attrition	PF Measurement	Outcome	Confounding	Statistical Analysis	Overall	Key Rationale
Vanderveken 2013 [11]	L	L	M	L	H	M	HIGH	Foundational study; OLW not formally tested as independent predictor; no multivariable adjustment; small cohort.
Mulholland and Dedhia 2020 [24]	M	M	H	H	H	L	MOD	Bespoke MA response grading; small *n* = 46; full-night efficacy PSG strength offsets some concerns.
Chao and Thaler 2020 [13]	M	L	M	H	H	M	HIGH	Differential outcome ascertainment between R/NR groups; complete-OLW subgroup *n* = 3.
Huyett 2021 [23]	L	L	L–M	H	M	L	MOD	Multicentre; 4 blinded reviewers (κ = 0.48); titration PSG primary outcome; multivariable adjustment performed.
Pordzik 2023 [25]	M	M	M	L	H	M	HIGH	*n* = 25 severely underpowered; no multivariable adjustment for BMI/AHI; full-night PSG is a methodological strength.
Nord 2024 [16]	M	M	H	M	H	M	HIGH	Single unblinded surgeon scored DISE; no multivariable adjustment; mixed PSG types.
Huyett 2024 [19]	M	M	M	M	M	L	MOD	Single surgeon scored DISE; titration PSG; partial registry overlap with Huyett 2021; high industry COI.
Vena 2025 [17]	L	L	M	M	L	L	MOD	Largest prospective cohort (*n* = 369); pre-specified multivariable regression adjusting for age, sex, BMI, baseline AHI, PSG type; single-surgeon DISE scoring without inter-rater reliability data; high industry COI.

Domain ratings: L = Low; M = Moderate; H = High; L–M = between Low and Moderate. Overall rating reflects a domain-weighted judgement, with study confounding and prognostic factor measurement weighted most heavily given the prognostic-review framing. “PF measurement” refers to prognostic factor measurement. Industry funding or author consultancy with the device manufacturer is recorded in the rationale column where present but is not a formal QUIPS domain.

## Data Availability

No new data were created or analyzed in this study. Data sharing is not applicable to this article.

## Supplementary Materials

The following supporting information can be downloaded at https://www.mdpi.com/article/10.3390/diagnostics16162529/s1, File S1: PRISMA 2020 checklist; Supplementary Table S1: Database Search Strategies; File S2: PROSPERO Registration PDF [29] was cited in Supplementary Materials.

## Abbreviations

The following abbreviations are used in this manuscript:

Abbreviation	Definition
AHI	Apnoea–Hypopnoea Index
AP	Anteroposterior
BMI	Body Mass Index
CCC	Complete Concentric Collapse
CENTRAL	Cochrane Central Register of Controlled Trials
CI	Confidence Interval
CPAP	Continuous Positive Airway Pressure
DISE	Drug-Induced Sleep Endoscopy
ESS	Epworth Sleepiness Scale
FDA	Food and Drug Administration
HGNS	Hypoglossal Nerve Stimulation
HNS	Hypoglossal Nerve Stimulation
HSAT	Home Sleep Apnoea Testing
HST	Home Sleep Test
ODI	Oxygen Desaturation Index
OPLW	Oropharyngeal Lateral Wall
OSA	Obstructive Sleep Apnoea
OLW	Oropharyngeal Lateral Wall
PSG	Polysomnography
PRISMA	Preferred Reporting Items for Systematic Reviews and Meta-Analyses
PROSPERO	International Prospective Register of Systematic Reviews
QUIPS	Quality in Prognosis Studies
SD	Standard Deviation
SPW	Supine Pharyngeal Width
tPSG	Titration Polysomnography
UAS	Upper Airway Stimulation
VOTE	Velum, Oropharyngeal Lateral Wall, Tongue Base, Epiglottis
