# Supplementary material for: Oropharyngeal Lateral Wall Collapse on Drug-Induced Sleep Endoscopy as a Predictor of Hypoglossal Nerve Stimulation Response in Obstructive Sleep Apnoea—A Systematic Review
Source: Diagnostics (Basel). 2026 Aug 11;16(16):2529. doi: 10.3390/diagnostics16162529 (PMC13511929; doi:10.3390/diagnostics16162529)
Supplement: Supplementary file 1 [file diagnostics-16-02529-s001.zip › File S2. PROSPERO Registration PDF.pdf]

Oropharyngeal lateral wall collapse on drug-induced sleep endoscopy as a predictor of hypoglossal nerve stimulation response in obstructive sleep apnoea: a systematic review

Sarah McBrinn, Aphrodite Iacovidou

Citation

Sarah McBrinn, Aphrodite Iacovidou. Oropharyngeal lateral wall collapse on drug-induced sleep endoscopy as a predictor of hypoglossal nerve stimulation response in obstructive sleep apnoea: a systematic review. Not yet published.

PREVIEW

REVIEW TITLE AND BASIC DETAILS

Review title

Oropharyngeal lateral wall collapse on drug-induced sleep endoscopy as a predictor of hypoglossal nerve stimulation response in obstructive sleep apnoea: a systematic review

Condition or domain being studied

Sleep Apnea; Obstructive Sleep Apnea Syndrome

Hypoglossal nerve implants to treat moderate-severe obstructive sleep apnoea

Rationale for the review

Hypoglossal nerve stimulation is an established treatment for moderate-to-severe obstructive sleep apnoea in patients intolerant of continuous positive airway pressure. Patient selection currently requires pre-operative drug-induced sleep endoscopy, with complete concentric collapse at the velopharynx representing the only formally accepted DISE-based contraindication. Oropharyngeal lateral wall collapse is a commonly observed DISE finding, but its prognostic significance for HNS outcomes remains contested. Published studies report conflicting findings, and no synthesis has specifically evaluated OLW collapse as a predictor of HNS response. Clarifying this relationship has direct implications for patient selection and surgical decision-making.Obstructive sleep apnoea (OSA) is a common condition associated with significant cardiovascular and neurocognitive morbidity. Continuous positive airway pressure (CPAP) remains the first-line treatment; however, long-term adherence is frequently suboptimal. Hypoglossal nerve stimulation (HNS) has emerged as an effective surgical treatment for selected patients with moderate-to-severe OSA who are intolerant of CPAP.

Despite increasing adoption, clinical response to HNS varies considerably between patients, highlighting the importance of appropriate patient selection. Current selection criteria are based on factors such as body mass index and findings from drug-induced sleep endoscopy, but the strength and consistency of evidence supporting these predictors remain unclear.

Although multiple studies have evaluated outcomes following HNS, there has been limited synthesis of patient-level predictors of treatment response across the literature. A systematic review is therefore required to identify and critically appraise factors associated with favourable outcomes, with the aim of improving patient selection and informing clinical practice.

Review objectives

To determine if oropharyngeal lateral wall collapse on pre-operative DISE can predict response to hypoglossal nerve stimulation in OSA.

Keywords

Obstructive sleep apnoea; Hypoglossal nerve stimulation; Upper airway stimulation; Patient selection; Predictors of response; Drug-induced sleep endoscopy; Body mass index; Sleep Surgery

Country

United Kingdom

ELIGIBILITY CRITERIA

Population

Included
Adults aged 18 years and over with moderate-to-severe obstructive sleep apnoea (AHI ≥15 events/hour) undergoing evaluation for hypoglossal nerve stimulation with any device (unilateral, bilateral, or proximal).

Adults ≥18 years with OSA (AHI ≥15/hr)

Pre-operative DISE performed and OLW findings reported

HNS implantation with any device

Quantitative association between OLW collapse and HNS outcome reported

Full-night non-titration follow-up sleep study

Prospective or retrospective cohort, case-control, or cross-sectional studies with outcome data

English

Excluded
Review articles, editorials, letters, conference abstracts without full text, case reports (n<5)

Non-DISE index test (PSG airflow morphology, MRI, cephalometry alone)

Follow-up based exclusively on titration polysomnography

No quantitative association between OLW findings and HNS outcome reported

Paediatric populations only

Intervention(s) or exposure(s)

Included
Presence of oropharyngeal lateral wall collapse (complete or partial) identified on pre-operative drug-induced sleep endoscopy using any validated classification system (VOTE, NOHL, or equivalent).

Comparator(s) or control(s)

Included
Absence of oropharyngeal lateral wall collapse on pre-operative DISE.

Study design

Both randomized and nonrandomized study types will be included.

Included
Randomised controlled trials, non-randomised interventional studies, prospective and retrospective cohort studies, and registry-based studies.

Excluded
Review articles, editorials, letters, conference abstracts without full text, case reports (n<5)

Non-DISE index test (PSG airflow morphology, MRI, cephalometry alone)

Follow-up based exclusively on titration polysomnography

No quantitative association between OLW findings and HNS outcome reported

Paediatric populations only

Context

Hypoglossal nerve stimulation is an established surgical treatment for selected patients with obstructive sleep apnoea who are intolerant of continuous positive airway pressure therapy. Its use is increasing internationally, including within healthcare systems such as the UK National Health Service. However, access remains limited and careful patient selection is required to optimise outcomes. This review is conducted in the context of evolving clinical pathways for sleep surgery and aims to inform decision-making regarding patient selection for hypoglossal nerve stimulation.

SIMILAR REVIEWS

Check for similar records already in PROSPERO

PROSPERO identified a number of existing PROSPERO records that were similar to this one (last check made on 21 March 2026). These are shown below along with the reasons given by that the review team for the reviews being different and/or proceeding.

- Hypoglossal Nerve Stimulation for the Treatment of Obstructive Sleep Apnea: A Systematic Review and Meta-Analysis [published 22 October 2025] [CRD420251173889]. The review was judged **not to be similar**
- Prognostic Indicators for Successful Hypoglossal Nerve Stimulation Therapy for Obstructive Sleep Apnea: A Systematic Review [published 24 February 2021] [CRD42021233060]. The review was acknowledged as **similar** but the authors opted to continue because *there are differences in intervention or comparator*, *the review uses improved methods*, *the review will be more up to date*
- How do specific collapse patterns identified during drug-induced sleep endoscopy(DISE) affect the success of upper airway stimulation(UAS) or hypoglossal nerve stimulation(HGNS) surgery in adults with obstructive sleep apnea(OSA)? [published 30 August 2025] [CRD420251115535]. The review was judged **not to be similar**

TIMELINE OF THE REVIEW

Date of first submission to PROSPERO
This record has not been submitted.

Review timeline
Start date: 21 March 2026. End date: 1 June 2026.

Date of registration in PROSPERO
This record has not been published.

AVAILABILITY OF FULL PROTOCOL

Availability of full protocol
A full protocol has been written and uploaded to PROSPERO. The protocol will be made available after the review is completed.

SEARCHING AND SCREENING

Search for unpublished studies
Only published studies will be sought.

Main bibliographic databases that will be searched
The main databases to be searched are *CENTRAL - Cochrane Central Register of Controlled Trials*, *Embase - Embase via Ovid* and *MEDLINE*.

Search language restrictions
The review will only include studies published in English.

Search date restrictions
There are no search date restrictions.

Other methods of identifying studies
Other studies will be identified by: *searching trial or study registers*.

Link to search strategy
A full search strategy has been uploaded to PROSPERO. The PDF may be accessed through this link https://www.crd.york.ac.uk/PROSPEROFILES/00f4bd393342a3ffc6ab518bae2f0b50.pdf.

Selection process
Studies will be screened independently by at least two people (or person/machine combination) with a process to resolve differences.

Other relevant information about searching and screening
None

DATA COLLECTION PROCESS

Data extraction from published articles and reports

Data will be extracted independently by at least two people (or person/machine combination) with a process to resolve differences.

Authors will not be contacted for further information.

Study risk of bias or quality assessment

Risk of bias will be assessed using:

QUIPS

Data will be assessed by one person (or a machine) and checked by at least one other person (or machine).

Additional information will **not** be sought from study investigators if required information is unclear or unavailable in the study publications/reports.

Reporting bias assessment

Risk of bias due to missing results will not be assessed

Certainty assessment

Certainty of findings will not be assessed

OUTCOMES TO BE ANALYSED

Main outcomes

Hypoglossal nerve stimulation surgical response, defined as a reduction in apnoea-hypopnoea index of ≥50% from pre-operative baseline and a post-treatment AHI of <20 events per hour (Sher criteria). Outcome must be assessed at minimum three months post-device activation using a full-night non-titration polysomnography or home sleep apnoea test with the device active.

Additional outcomes

Absolute AHI reduction from baseline to follow-up

Oxygen desaturation index (ODI) reduction

Epworth Sleepiness Scale (ESS) score improvement

Surgical response by alternative criteria where reported (AHI <15 events/hour; AHI <10 events/hour; AHI reduction ≥50% without absolute threshold)

Odds ratio or relative risk of surgical response by OLW collapse status

Independent predictive value of OLW collapse on multivariate analysis adjusted for clinical covariates

PLANNED DATA SYNTHESIS

Strategy for data synthesis

A narrative synthesis will be performed as the primary approach, structured by degree of OLW collapse (complete versus partial versus any) and by whether studies report univariate or multivariate adjusted associations. Given anticipated heterogeneity in DISE classification systems, HNS device types, response definitions, and follow-up methodology, meta-analysis will only be performed where three or more studies report sufficiently comparable effect estimates — specifically odds ratios or relative risks for surgical response by OLW collapse status using the Sher criteria with full-night non-titration follow-up sleep studies. Where meta-analysis is performed, a random-effects model using the DerSimonian-Laird method will be applied. Statistical heterogeneity will be assessed using the I^2 statistic; I^2 >50% will be considered substantial. Pre-specified subgroup analyses will be conducted by HNS device type and by response definition. Funnel plot asymmetry will be assessed if ten or more studies are included in a meta-analysis.

CURRENT REVIEW STAGE

Stage of the review at this submission

Review stage	Started	Completed
Pilot work	✓	
Formal searching/study identification	✓	
Screening search results against inclusion criteria	✓	
Data extraction or receipt of IPD		
Risk of bias/quality assessment		
Data synthesis		

Review status
The review is currently planned or ongoing.

Publication of review results
Results of the review will be published in English.

REVIEW AFFILIATION, FUNDING AND PEER REVIEW

Review team members
Ms Sarah McBrinn (review guarantor and contact) ORCID: 0009-0004-3876-3924. Imperial NHS Foundation Trust. England.

No conflict of interest declared.

Ms Aphrodite Iacovidou. Imperial NHS Foundation Trust. England.

No conflict of interest declared.

Named contact
Ms Sarah McBrinn (sarah.mcbrinn1@nhs.net). ORCID: 0009-0004-3876-3924. Imperial NHS Foundation Trust. England.

Review affiliation
Imperial College NHS Foundation Trust

Funding source
Review has no funding and no agreed support from an academic institution and is done in authors' own time.

Peer review
There has been no peer review of this planned review.

ADDITIONAL INFORMATION

Review conflict of interest
Declared individual interests are recorded under team member details.. No additional interests are recorded for this review.

Medical Subject Headings
Endoscopy; Hypoglossal Nerve; Sleep Apnea, Obstructive

PROSPERO version history
No preview available

Disclaimer
The content of this record displays the information provided by the review team. PROSPERO does not peer review registration records or endorse their content.

PROSPERO accepts and posts the information provided in good faith; responsibility for record content rests with the review team. The guarantor for this record has affirmed that the information provided is truthful and that they understand that deliberate provision of inaccurate information may be construed as scientific misconduct.

PROSPERO does not accept any liability for the content provided in this record or for its use. Readers use the information provided in this record at their own risk.

Any enquiries about the record should be referred to the named review contact
