# Supplementary material for: Oropharyngeal Lateral Wall Collapse on Drug-Induced Sleep Endoscopy as a Predictor of Hypoglossal Nerve Stimulation Response in Obstructive Sleep Apnoea—A Systematic Review
Source: Diagnostics (Basel). 2026 Aug 11;16(16):2529. doi: 10.3390/diagnostics16162529 (PMC13511929; doi:10.3390/diagnostics16162529)
Supplement: Supplementary file 1 [file diagnostics-16-02529-s001.zip › Supplementary_Table_S1.pdf]

Supplementary Table S1 — Database Search Strategies

Oropharyngeal Lateral Wall Collapse on Drug-Induced Sleep Endoscopy as a Predictor of Hypoglossal Nerve Stimulation Response in Obstructive Sleep Apnoea: A Systematic Review

McBrinn S, Skan O, Iacovidou A | PROSPERO: CRD420261347436

Search 1: MEDLINE via PubMed — Original Search

Date searched: 21 March 2026 | Records retrieved: 372

Search terms combined controlled vocabulary (MeSH) with free-text terms relating to hypoglossal nerve stimulation, obstructive sleep apnoea, drug-induced sleep endoscopy, and treatment response. No date restrictions or language filters were applied.

```
(
  "Inspire"[tiab] OR "upper airway stimulation"[tiab]
  OR "hypoglossal nerve stimulat*"[tiab]
  OR "hypoglossal nerve stimulation"[MeSH]
  OR "implantable neurostimulat*"[tiab]
  OR "UAS"[tiab] OR "HNS"[tiab]
)
AND
(
  "obstructive sleep apn*"[tiab]
  OR "sleep disordered breathing"[tiab]
  OR "sleep-disordered breathing"[tiab]
  OR "OSA"[tiab] OR "OSAHS"[tiab]
  OR "obstructive sleep apnea"[MeSH]
)
AND
(
  "drug-induced sleep endoscopy"[tiab]
  OR "drug induced sleep endoscopy"[tiab]
  OR "DISE"[tiab]
  OR "sleep endoscopy"[tiab]
  OR "sedation endoscopy"[tiab]
  OR "pharmacological sleep endoscopy"[tiab]
)
AND
(
  "predict*"[tiab] OR "responder*"[tiab]
  OR "non-responder*"[tiab] OR "nonresponder*"[tiab]
  OR "treatment response"[tiab]
  OR "surgical outcome*"[tiab]
  OR "patient selection"[tiab]
  OR "therapy success"[tiab] OR "therapy failure"[tiab]
  OR "success rate"[tiab]
)
```

Records retrieved: 372

Search 2: MEDLINE via PubMed — Supplementary DISE-Specific Search

Date searched: 21 March 2026 | Records retrieved: 246

A supplementary search was conducted with expanded DISE-specific terminology, including collapse pattern descriptors and alternative device names, to capture studies not retrieved by the original search.

```
(
```

```
  "Inspire"[tiab] OR "Genio"[tiab] OR "aura6000"[tiab]
  OR "upper airway stimulation"[tiab]
  OR "hypoglossal nerve stimulat*"[tiab]
  OR "hypoglossal nerve stimulation"[MeSH]
  OR "implantable neurostimulat*"[tiab]
  OR "UAS"[tiab] OR "HNS"[tiab]
)
AND
(
  "obstructive sleep apn*"[tiab]
  OR "sleep disordered breathing"[tiab]
  OR "sleep-disordered breathing"[tiab]
  OR "OSA"[tiab] OR "OSAHS"[tiab]
  OR "obstructive sleep apnea"[MeSH]
)
AND
(
  "VOTE"[tiab] OR "NOHL"[tiab]
  OR "lateral wall collapse"[tiab]
  OR "oropharyngeal lateral wall"[tiab]
  OR "lateral pharyngeal wall collapse"[tiab]
  OR "tongue base collapse"[tiab]
  OR "base of tongue collapse"[tiab]
  OR "retropalatal collapse"[tiab]
  OR "velopharyngeal collapse"[tiab]
  OR "epiglottic collapse"[tiab]
  OR "supraglottic collapse"[tiab]
  OR "anteroposterior collapse"[tiab]
  OR "concentric collapse"[tiab]
  OR "multilevel collapse"[tiab]
  OR "collapse pattern*"[tiab]
  OR "airflow shape"[tiab]
  OR "pharyngeal collapse"[tiab]
  OR "palatal collapse"[tiab]
)
AND
(
  "predict*"[tiab] OR "responder*"[tiab]
  OR "non-responder*"[tiab] OR "nonresponder*"[tiab]
  OR "treatment response"[tiab]
  OR "surgical outcome*"[tiab]
  OR "patient selection"[tiab]
  OR "therapy success"[tiab] OR "therapy failure"[tiab]
  OR "success rate"[tiab]
)
```

Records retrieved: 246

Search 3: EMBASE via Ovid

Date searched: 23 March 2026 | Records retrieved: 122

```
1.  (hypoglossal nerve stimulat* OR upper airway stimulat*
    OR inspire OR UAS OR HNS
    OR implantable neurostimulat*).ti,ab.
2.  exp hypoglossal nerve stimulation/
3.  1 OR 2
4.  (obstructive sleep apnea OR obstructive sleep apnoea
    OR OSA OR OSAHS OR sleep disordered breathing
    OR sleep-disordered breathing).ti,ab.
5.  exp sleep apnea obstructive/
6.  4 OR 5
```

```
7.   (drug-induced sleep endoscopy OR drug induced sleep endoscopy
     OR DISE OR sleep endoscopy OR sedation endoscopy).ti,ab.
8.   (VOTE OR NOHL OR collapse pattern* OR lateral wall collapse
     OR oropharyngeal lateral wall OR tongue base collapse
     OR retropalatal collapse OR epiglottic collapse
     OR concentric collapse OR anteroposterior collapse
     OR multilevel collapse OR palatal collapse
     OR airflow shape OR pharyngeal collapse).ti,ab.
9.   7 OR 8
10.  (predict* OR responder* OR nonresponder*
     OR treatment response OR surgical outcome*
     OR patient selection OR therapy success
     OR therapy failure OR success rate).ti,ab.
11.  3 AND 6 AND 9 AND 10
12.  limit 11 to human
13.  limit 12 to english language
```

Records retrieved: 122

Search 4: Cochrane Central Register of Controlled Trials (CENTRAL)

Date searched: 23 March 2026 | Records retrieved: 11

```
#1   "hypoglossal nerve stimulation" OR "upper airway stimulation"
     OR "Inspire" OR "HNS" OR "UAS"
#2   "obstructive sleep apnea" OR "obstructive sleep apnoea" OR "OSA"
#3   "drug-induced sleep endoscopy" OR "DISE" OR "sleep endoscopy"
     OR "VOTE" OR "lateral wall collapse"
     OR "oropharyngeal lateral wall" OR "collapse pattern"
#4   "treatment response" OR "surgical outcome"
     OR "patient selection" OR "predictor"
#5   #1 AND #2 AND #3 AND #4
```

Records retrieved: 11

Search 5: ClinicalTrials.gov

Date searched: 23 March 2026 | Records retrieved: 2

```
Search terms: "hypoglossal nerve stimulation" AND "sleep endoscopy"
```

Two registered trials were identified. Both were actively recruiting with no results available at the time of search and were therefore excluded from the primary synthesis.

Records retrieved: 2 (excluded — no results available)

Summary of Search Results

Database	Date	Records	Included
MEDLINE via PubMed (original)	21 Mar 2026	372	Yes
MEDLINE via PubMed (supplementary)	21 Mar 2026	246	Yes

Database	Date	Records	Included
EMBASE via Ovid	23 Mar 2026	122	Yes
Cochrane CENTRAL	23 Mar 2026	11	Yes
ClinicalTrials.gov	23 Mar 2026	2	No — no results
**Total identified**		**751**	
**After deduplication (Rayyan)**		**688**	Screened

Note: Duplicate removal was performed using Rayyan systematic review software. The two ClinicalTrials.gov records represented actively recruiting trials with no published results and were excluded from the primary synthesis.
